# Malignant Mesothelioma Diagnosed From a Spermatic Cord Lesion After Inconclusive Thoracoscopy: A Case With Concomitant Pleural and Peritoneal Involvement

**DOI:** 10.7759/cureus.109236

**Published:** 2026-05-19

**Authors:** Naoko Hamazaki, Sayaka Fujimoto, Hiroko Jinno, Nao Miyazaki, Takahiro Masuda, Hiroki Sato, Yoko Kida, Masatsugu Yamamoto, Kimihide Tada

**Affiliations:** 1 Department of Internal Medicine, Mitsubishi Kobe Hospital, Kobe, JPN; 2 Department of Respiratory Medicine, Toyooka Public Hospital, Toyooka, JPN; 3 Department of Respiratory Medicine, Doai Memorial Hospital, Tokyo, JPN; 4 Department of Respiratory Medicine, Kobe University Hospital, Kobe, JPN; 5 Department of Respiratory Medicine, Kobe City Nishi-Kobe Medical Center, Kobe, JPN

**Keywords:** diagnostic challenge, laparoscopy, malignant mesothelioma, spermatic cord, thoracoscopy

## Abstract

Malignant mesothelioma typically arises in the pleura or peritoneum; however, involvement of the spermatic cord is extremely rare. Here, we report a case in which a definitive diagnosis was achieved through evaluation of a spermatic cord lesion after an inconclusive thoracoscopic biopsy.

A 69-year-old man presented with dyspnea and right inguinal swelling. Imaging revealed a right pleural effusion, peritoneal nodules, and increased fluorodeoxyglucose uptake in the diaphragm and bilateral inguinal canals. Thoracoscopy under local anesthesia revealed a diaphragmatic mass; however, the biopsy specimen was insufficient for a definitive diagnosis.

Subsequent right orchiectomy and laparoscopic examination revealed disseminated peritoneal nodules and a spermatic cord tumor. Histopathological examination of spermatic cord specimens showed invasive epithelioid malignant mesothelioma.

This case highlights the limitations of small thoracoscopic biopsy specimens and underscores the importance of selecting the appropriate biopsy sites and techniques when mesothelioma is suspected.

## Introduction

Malignant mesothelioma is an aggressive neoplasm arising from mesothelial cells, most commonly involving the pleura and peritoneum. In contrast, involvement of the spermatic cord is extremely rare [1]. There is a well-established causal relationship between asbestos exposure and malignant mesothelioma. The disease carries a poor prognosis, with a median survival of approximately 9-12 months from the time of presentation [2].

Diagnosis typically requires histopathological confirmation with evidence of stromal invasion in addition to immunohistochemical findings [3]. However, small or superficial biopsy specimens may limit diagnostic accuracy and make differentiation from reactive mesothelial proliferations challenging.

Thoracoscopy under local anesthesia is widely used due to its minimally invasive nature and high diagnostic yield [4]. Nevertheless, its limitations have been reported, particularly when biopsy specimens lack sufficient depth to demonstrate stromal invasion [5].

Furthermore, in cases of multisite involvement, selecting an appropriate biopsy site is crucial to obtain adequate tissue for diagnosis. However, evidence guiding the selection of the optimal biopsy site for such complex presentations remains limited.

We report a rare and diagnostically challenging case of malignant mesothelioma involving the pleura, peritoneum, and spermatic cord, in which a definitive diagnosis was achieved only after surgical sampling of a spermatic cord lesion, highlighting the importance of biopsy site selection.

## Case presentation

A 69-year-old man presented with dyspnea on exertion and a right-sided inguinal mass. His medical history included an endoscopic submucosal dissection for gastric cancer and hyperuricemia. He had no history of smoking or exposure to occupational dust or asbestos.

Chest radiography and computed tomography revealed a right pleural effusion without obvious masses or pleural thickening (Figure 1). Fluorodeoxyglucose positron emission tomography/computed tomography (FDG-PET/CT) demonstrated increased uptake in a nodule above the diaphragm, multiple peritoneal nodules, and the right inguinal canal, with mild uptake in the left inguinal canal. Ascites was also present (Figure 2).

**Figure 1:**
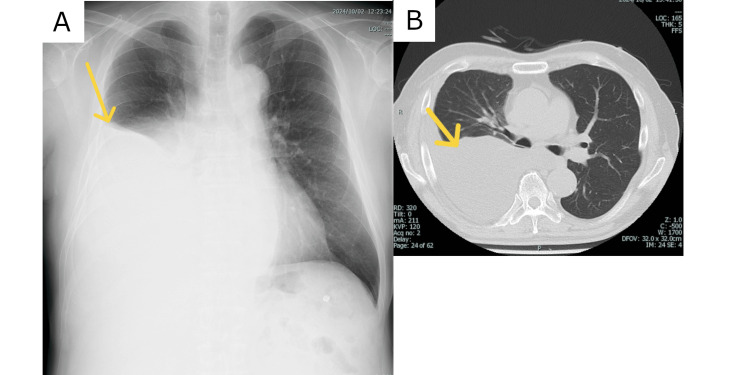
A) Chest radiograph showing a right pleural effusion; B) Chest computed tomography (CT) showing a right pleural effusion

**Figure 2:**
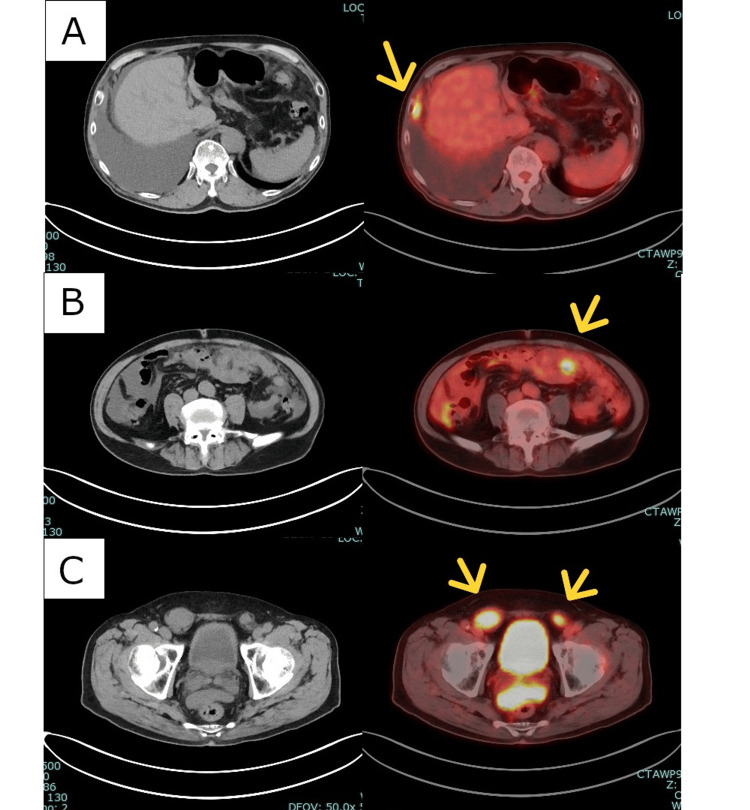
Positron emission tomography–computed tomography (PET–CT) images showing abnormal fluorodeoxyglucose uptake in the diaphragm (A), peritoneum (B), and spermatic cord (C)

Thoracentesis yielded bloody pleural fluid with elevated hyaluronic acid levels; however, the cytology was negative for malignancy.

Thoracoscopy under local anesthesia revealed a solitary mass above the diaphragm without diffuse pleural abnormalities (Figure 3A). Biopsy specimens showed papillary proliferation of tumor cells positive for D2-40, WT-1, and calretinin (Figure 4A, 4B). However, definitive stromal invasion was not identified, and differentiation from reactive mesothelial hyperplasia was inconclusive.

**Figure 3:**
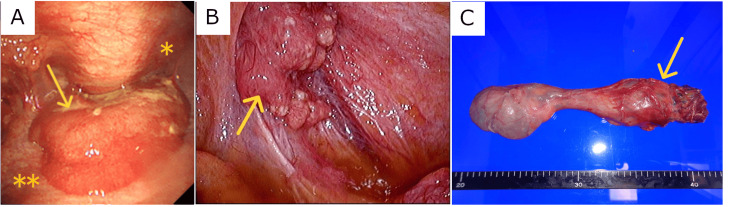
A) Thoracoscopic view showing a mass located between the anterior pleural wall and the diaphragm (*pleural wall, **diaphragm); B) Laparoscopic view showing exposure of a tumor arising from the spermatic cord (arrow); C) Gross specimen of the resected spermatic cord tumor (arrow)

**Figure 4:**
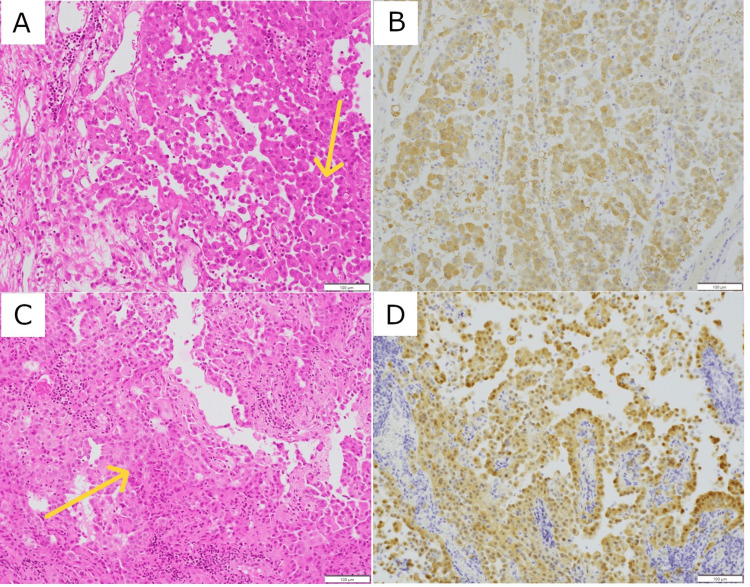
A) Histopathological findings of the pleural lesion showing papillary structures (H&E staining); B) Immunohistochemical staining showing tumor cells positive for calretinin in the pleural lesion; C) Histopathological findings of the spermatic cord tumor showing a nesting growth pattern; D) Immunohistochemical staining showing tumor cells positive for calretinin in the spermatic cord tumor

Given strong FDG uptake in the inguinal region, the patient underwent right orchiectomy with laparoscopic exploration. Laparoscopy revealed multiple peritoneal nodules and ascitic fluid (Figure 3B, 3C).

Histopathological examination of the spermatic cord tumor revealed solid proliferation of atypical cells with eosinophilic cytoplasm, nuclear pleomorphism, and clear invasion of the surrounding adipose tissue. Immunohistochemical analysis revealed positivity for calretinin, WT-1, and membranous epithelial membrane antigen (EMA) expression and negativity for desmin (Figure 4C, 4D). These findings confirmed a diagnosis of epithelioid malignant mesothelioma.

The disease was considered unresectable because of its extensive involvement. The patient was administered cisplatin and pemetrexed. The disease remained stable per the response evaluation criteria in solid tumors (RECIST) criteria, and no new lesions were observed during follow-up.

## Discussion

Malignant mesothelioma is an aggressive tumor arising from mesothelial cells, most commonly involving the pleura and peritoneum, while involvement of other sites, including the spermatic cord, accounts for less than 1% of cases [1]. Asbestos exposure is a well-established risk factor; however, some patients have no identifiable exposure history [6]. In the present case, no history of asbestos exposure was identified.

Thoracoscopy under local anesthesia is widely used as a minimally invasive diagnostic procedure for patients with pleural effusion, allowing direct visualization and biopsy of pleural lesions [4]. Although it generally provides a high diagnostic yield, limitations have been reported, particularly when biopsy specimens are small or superficial [5]. In the present case, immunohistochemical findings suggested mesothelioma; however, definitive stromal invasion was not identified, making differentiation from reactive mesothelial hyperplasia difficult. Since the diagnosis of mesothelioma requires histological evidence of invasion in addition to immunohistochemical findings [3], small biopsy specimens may be insufficient for a definitive diagnosis. Previous studies have also reported that limited tissue sampling can reduce diagnostic accuracy [5]. In this case, thoracoscopic biopsy specimens were confined to superficial layers, highlighting the limitations of small biopsy samples.

In contrast, the spermatic cord lesion obtained via high orchiectomy demonstrated clear stromal invasion, leading to a definitive diagnosis of malignant mesothelioma. Compared to thoracoscopic biopsy, surgical sampling allows the acquisition of larger and deeper tissue specimens, enabling more reliable histopathological evaluation.

Mesothelioma is known to exhibit local progression as well as dissemination and metastasis, and cases involving multiple sites, including the pleura, peritoneum, and spermatic cord, have been reported [7]. In particular, malignant mesothelioma of the spermatic cord is exceedingly rare, occurring either as a primary tumor or as a secondary extension from peritoneal disease [8]. Therefore, the identification of a spermatic cord lesion may warrant consideration of concomitant peritoneal involvement. The clinical and pathological features of these reported cases, including the present case, are summarized in Table 1. In the present case, FDG-PET/CT demonstrated uptake in both inguinal regions, suggesting possible dissemination or extension from peritoneal disease. However, the metastatic patterns of mesothelioma have not been fully elucidated, and the frequency and risk factors for spread to other mesothelial sites remain unclear [9]. The histopathological similarity among lesions in the pleura, peritoneum, and spermatic cord suggests that these lesions likely represent manifestations of a single disease process.

**Table 1:** Comparison of malignant mesothelioma cases with spermatic cord or multisite involvement

Reference	Age	Asbestos exposure	Site of involvement	Histologic type	Definitive diagnostic site
Present case	69	No	Pleura, peritoneum, and spermatic cord	Epithelioid	Spermatic cord
Tsuruga et al. [7]	72	Yes	Pleura, peritoneum, and spermatic cord	Epithelioid	Spermatic cord
Kobayashi et al. [10]	82	No	Spermatic cord (in situ)	Epithelioid	Spermatic cord
Park et al. [11]	65	Yes	Spermatic cord and peritoneum	Biphasic	Spermatic cord and peritoneal nodules
Meng et al. [8]	45	No	Spermatic cord	Epithelioid	Spermatic cord
Meng et al. [8]	68	No	Peritoneum and spermatic cord	Biphasic	Spermatic cord

This case highlights the importance of selecting appropriate biopsy sites and techniques in patients with suspected mesothelioma, particularly when initial biopsy results are inconclusive. When minimally invasive procedures fail to provide sufficient diagnostic information, more invasive surgical approaches should be considered to obtain adequate tissue for definitive diagnosis.

## Conclusions

Malignant mesotheliomas may be difficult to diagnose when biopsy specimens are limited. This case demonstrates that surgical sampling from an alternative lesion, such as the spermatic cord, can be crucial for establishing a definitive diagnosis. Careful selection of biopsy sites is essential in diagnostically challenging cases.
